# Viral Escape Mutant Epitope Maintains TCR Affinity for Antigen yet Curtails CD8 T Cell Responses

**DOI:** 10.1371/journal.pone.0149582

**Published:** 2016-02-25

**Authors:** Shayla K. Shorter, Frederick J. Schnell, Sean R. McMaster, David F. Pinelli, Rakieb Andargachew, Brian D. Evavold

**Affiliations:** Department of Microbiology and Immunology, Emory University, Atlanta, Georgia, United States of America; Maisonneuve-Rosemont Hospital, CANADA

## Abstract

T cells have the remarkable ability to recognize antigen with great specificity and in turn mount an appropriate and robust immune response. Critical to this process is the initial T cell antigen recognition and subsequent signal transduction events. This antigen recognition can be modulated at the site of TCR interaction with peptide:major histocompatibility (pMHC) or peptide interaction with the MHC molecule. Both events could have a range of effects on T cell fate. Though responses to antigens that bind sub-optimally to TCR, known as altered peptide ligands (APL), have been studied extensively, the impact of disrupting antigen binding to MHC has been highlighted to a lesser extent and is usually considered to result in complete loss of epitope recognition. Here we present a model of viral evasion from CD8 T cell immuno-surveillance by a lymphocytic choriomeningitis virus (LCMV) escape mutant with an epitope for which TCR affinity for pMHC remains high but where the antigenic peptide binds sub optimally to MHC. Despite high TCR affinity for variant epitope, levels of interferon regulatory factor-4 (IRF4) are not sustained in response to the variant indicating differences in perceived TCR signal strength. The CD8+ T cell response to the variant epitope is characterized by early proliferation and up-regulation of activation markers. Interestingly, this response is not maintained and is characterized by a lack in IL-2 and IFNγ production, increased apoptosis and an abrogated glycolytic response. We show that disrupting the stability of peptide in MHC can effectively disrupt TCR signal strength despite unchanged affinity for TCR and can significantly impact the CD8+ T cell response to a viral escape mutant.

## Introduction

Although there are many contributing arms of an effective immune response, T cells are one of the most significant players. T cell receptors display an impressive breadth of specificity for a wide variety of antigens owed largely to the process of T cell development in the thymus where VDJ rearrangement can generate a diverse repertoire [1]. Engagement of TCR with cognate peptide: MHC initiates downstream signaling cascades leading to up-regulation of activation markers, cytokine production and proliferation [2, 3]. While full T cell activation is the result of a combination of signals derived from co-stimulation and cytokine signals, the initial TCR recognition of antigen is a critical aspect of this process and a determinate of T cell fate [4, 5]. Thus, modifications that affect the ability of TCR to bind peptide:MHC as in the case of altered peptide ligands (APL), can dramatically impact an ensuing T cell response. The effect of APLs on T cell function have been characterized using peptide variants with mutated TCR contact residues [6, 7]. These studies demonstrated that TCR affinity for APL directly correlated with T cell function, with high affinity peptides defined as agonists that induced maximal T cell activation. Mutations that result in suboptimal binding to TCR have been shown to limit downstream signaling and gene expression involved in activation, proliferation and development of effector function. The result is a variety of outcomes including partial activation, T cell antagonism or anergy, where T cell activation is blocked by the initiation of negative signaling cascades [8]. These studies have led to a variety of therapeutics for anti-tumor and autoimmune responses with mixed success [9, 10]. Most include vaccination with altered peptide ligand epitopes and/or engineering T cells to express receptors with supraoptimal affinity for peptide:MHC in effort to enhance or abrogate T cell responses [11–13].

The study of the affinity parameters that govern a productive recognition event is of great interest as these fundamental mechanisms inform our understanding of immune responses ranging from autoimmunity to viral infection. Though responses to antigens that have differential binding affinities to TCR have been studied extensively, understanding the impact of disrupting binding to MHC has been highlighted to a lesser extent. Like their APL counterparts, recognition of MHC variant peptides (MVP) can alter T cell phenotype, but in this case the TCR affinity for antigenic pMHC is likely maintained with the parameter of pMHC stability driving the sub-optimal triggering. For instance, we have previously shown an MHC variant peptide of myelin oligodendrocyte (MOG) can anergize polyclonal encephalitogenic T cells by triggering negative signaling mediated by Src homology tyrosine phosphatase (SHP-1)[14]. This suggests that unlike APLs that have effects on clonal TCRs and in MS patients caused exacerbation of clinical symptoms [15], MVPs could be more effective in therapeutically abrogating polyclonal T cell mediated autoimmune disease. The advantage of manipulating pMHC stability to alter T cell activation state allows for a broader impact on polyclonal T cell responses rather than a single clone as would be in the case of APL recognition. While MVP-induced unresponsiveness in T cells may be beneficial in the context of autoimmunity, we hypothesized that a similar mechanism could be exploited by pathogens to subvert the T cell response.

In our study, we describe a strategy by which recognition of a lymphocytic choriomeningitis (LCMV) viral variant epitope with diminished affinity for MHC leads to a subpar outcome despite having similar TCR affinity as the wildtype epitope. This previously identified viral variant (henceforth referred to as 35A) contains a single amino acid mutation in position 35, changing a valine to alanine and resulting in a considerably lower affinity for H-2D^b^ [16]. We have also previously shown that the 35A epitope is not ignored but that this viral variant recognition results in modification of the SHP-1 tyrosine phosphatase which plays a role in mediating negative regulation of TCR signaling [17]. Here, we extend our findings to show that CD8+ T cells from the P14 TCR transgenic mice specific for the original GP33 epitope can recognize the mutated epitope with the same two dimensional (2D) affinity. Although TCR affinity tends to correlate with functional outcome for APL [18, 19], here recognition of the viral variant leads to diminished interferon regulatory factor 4 (IRF4) expression indicating that T cells interpret variant 35A as weak stimulus. Importantly, the 35A:D^b^ epitope rapidly decays displaying a half-life of 3 hours whereas the native GP33 peptide has a half-life of >5 hours. This high affinity TCR recognition of a less stable complex results in an early proliferative response and up-regulation of activation markers. This initial response to the variant is not sustained and instead culminates in abortive proliferation and death of responding effectors. The blunted response is characterized by diminished production of IL-2 and IFNγ as well as a decreased glycolytic response. We show a model in which disruption of peptide stability on the MHC leads to abrogation of the CD8+ T cell response to an LCMV viral escape mutant.

## Results

### Recognition of 35A epitope results in decreased TCR signal strength despite unchanged TCR affinity

**Fig 1:**
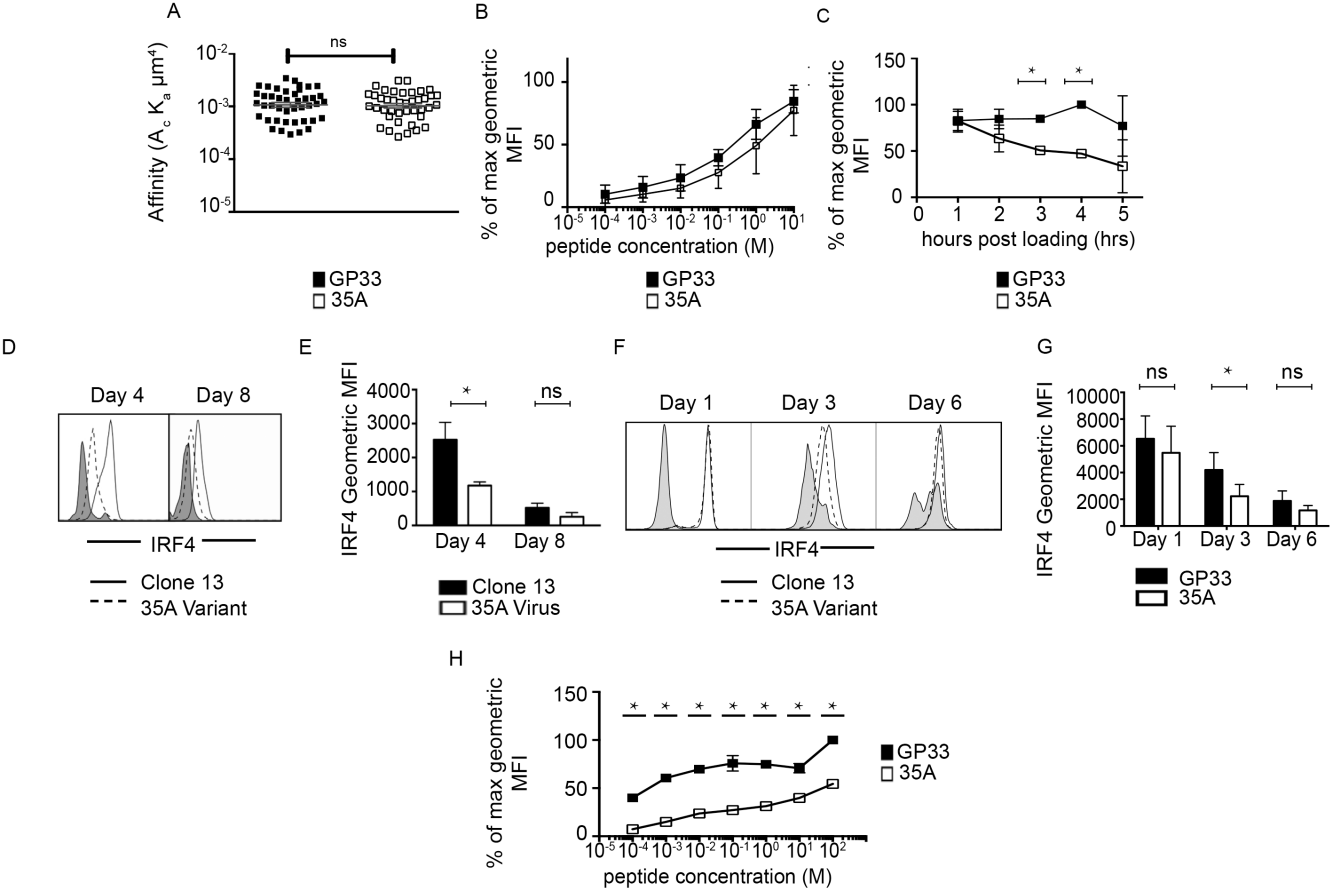
Variant peptide 35A maintains the same affinity for TCR as native GP33 epitope despite MHC instability and decreased IRF4 expression. (A) Affinity of GP33 and 35A epitope using the two-dimensional (2D) micropipette adhesion system. Average of 5 independent experiments. Total of 43 pairs tested for GP33 and 41 pairs for 35A. p = 0.775 n.s. (two tailed unpaired T test) (B) Stability of peptide on MHC was measured using an RMAS assay by loading different concentrations of peptide on empty class I and assaying surface expression of H-2D^b^ after 30 minutes post loading. Average of two independent experiments. (C) 10μm of each peptide is loaded onto RMAS cells and the gMFI of H-2D^b^ is measured over time to assess peptide stability and half life. Average of 3 independent experiments. *p<0.05 (Two way ANOVA Holm-Sidak) (D) and (E) 10^5^ P14 CD8+ T cells were adoptively transferred into congenic hosts and infected with 2 x10^3^ PFU Clone 13 or 35A variant. At 4 and 8 days post infection, spleens were harvested analyzed by flow cytometry for IRF4 expression on donor cells. Average of 3–4 independent experiments with 3–4 mice pooled per group. *p<0.05 (Paired t test)(F) and (G) P14 CD8 T cells were stimulated *in vitro* with 10μm wildtype GP33 peptide or variant 35A peptide and harvested at indicated time points and analyzed by flow cytometry for IRF4 expression. Average of 3–4 independent experiments. *p<0.05 (Paired t test) (H) P14 T cells were stimulated with GP33 or 35A peptide over a range of doses and analyzed for IRF4 expression at day 3. Data shown is the average of 2 independent experiments. *p<0.05 (Multiple t tests) Gray shaded histograms refer to cells isolated from uninfected control hosts or unstimulated cells. Error bars on all plots indicate ± SEM

Common methods of measuring affinity or avidity, by surface plasmon resonance (SPR) or tetramer fall-off assays, respectively have yielded much information on the kinetics and nature of TCR binding to peptide:MHC (pMHC). While affinity is generally accepted as a reliable predictor of functional outcome, there are cases that do not fit this predictive model. Measuring two-dimensional (2D) affinity using the micropipette adhesion frequency assay has been shown to more closely correlate with potency of T cells responses for both CD8 and CD4 T cells [18, 20–22]. Using this system, we measured the 2D affinity of P14 TCR transgenic CD8+ T cells for wildtype GP33 and mutant 35A epitope and found no significant difference (Fig 1A). This 2D affinity is also consistent with reports of identical avidity between the GP33 and 35A epitopes using pMHC tetramers [23]. As affinity is the outcome of the on rate and off rate of binding, it does not inform on the stability or half-life of the antigenic complex. Functional outcome is also dependent on the interaction between antigen and MHC, so we used the RMA-S MHC stability assay to determine the stability and half-life of the peptides on H-2D^b^. Both wildtype GP33 peptide and 35A peptide were able to stabilize the expression of H-2D^b^ on the surface of the RMA-S cells across a range of doses (Fig 1B). However, mutant 35A epitope was highly unstable, as even at the highest dose that gave equal loading on MHC (10μm), the mutant 35A rapidly decayed with a half-life of ~3 hours while the wildtype GP33 epitope had a half-life of >5 hours (Fig 1C).

Given these binding parameters, we wanted to investigate how T cells would interpret an antigen that maintains high TCR affinity and tetramer avidity but fails to stably bind MHC with long half-life as comparable to wildtype. Using D^b^ restricted P14 TCR transgenic CD8+ T cells, we measured IRF4 levels in response to stimulation with both the wildtype GP33 epitope and the 35A epitope. IRF4 is a transcription factor involved in regulating various aspects of lymphocyte differentiation and expansion and has also been shown to correlate with TCR affinity for antigen [24–26]. Interestingly, while P14 T cells responding to 35A virus *in vivo* upregulated IRF4 expression at day 4 post infection, this expression was significantly less than in the response to Clone 13 infection (Fig 1D and 1E). This pattern of expression is also demonstrated *in vitro* as 35A stimulated cells expressed less IRF4 on day 3 (Fig 1F and 1G). It should be noted however that within 24 hours of stimulation *in vitro*, IRF4 expression in both GP33 and 35A primed cells is comparable (Fig 1G). This suggests that T effectors can initially be triggered by 35A but the MHC instability causes IRF4 expression to be prematurely downregulated. This pattern can be observed over a range of peptide doses (Fig 1H), with 35A peptide stimulated cells expressing significantly less IRF4 at equivalent doses of peptide. While IRF4 has been described as a metric for TCR affinity for antigen, we found that despite similar affinity for antigen, 35A recognition resulted in decreased maintenance of IRF4 up-regulation. In this case, IRF4 expression does not exclusively readout TCR affinity for antigen, but the overall signal potency perceived by the TCR which is determined by both TCR affinity and peptide stability on MHC.

### Recognition of mutant epitope results in activation and abortive proliferation

**Fig 2:**
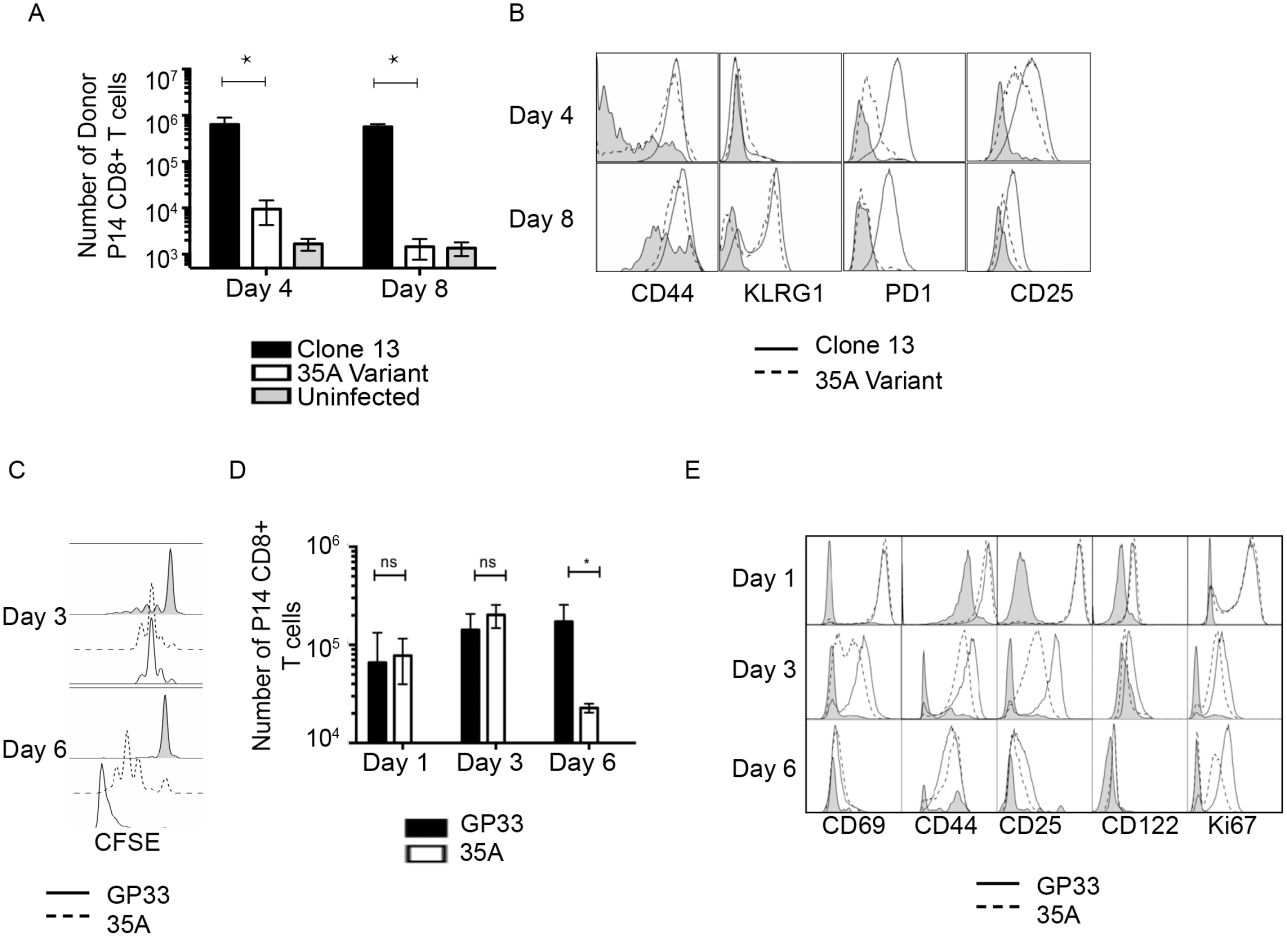
Recognition of 35A induces activation and proliferation of P14 CD8+ T cells without accumulation. (A) 10^5^ P14 Thy1.1 CD8+ T cells were adoptively transferred into C57BL/6 hosts and infected one day later with 2 x10^3^ PFU Clone 13 or 35A variant virus i.v. At 4 and 8 days post infection, spleens were harvested and analyzed for recovery of donor CD8+ T cells (A) and phenotypic analysis (B). Data from 3–5 experiments shown. n = 2–3 mice per group. *p<0.05 (2 way ANOVA multiple comparisons). Error bars indicate ± SEM. (C) P14 T cells were stimulated *in vitro* with 10μm GP33 or 35A peptide and harvested at day 3 or 6 to evaluate division by CFSE (C) total CD8+ T cell expansion (D) and phenotypic analysis (E). Representative data is shown in (C and E) or average of 3 experiments shown in (D) with samples run in duplicate for total cell number counts. *p<0.05 (2 way ANOVA multiple comparisons). Error bars indicate ± SEM. Gray shaded histograms refer to cells isolated from uninfected control hosts or unstimulated cells.

We sought to examine whether the TCR signal derived from recognition of 35A was of sufficient strength to sustain T cell activation and expansion *in vivo*. We adoptively transferred 10^5^ P14 transgenic T cells into congenic hosts and infected mice with a chronic LCMV strain, Clone 13, which contains the wild type GP33 epitope or the Clone 13 based variant virus 35A [27] (Fig 2A). Since we observed high mortality in animals infected with the traditional 2 x 10^6^ PFU dose of Clone 13, we reduced the dose to 2 x10^3^ PFU for each virus. By day 4 post infection, P14 T cells expanded in response to both viruses, albeit to a lesser extent to the variant virus (Fig 2A). As early as day 4 post infection, donor cells responding to Clone 13 not only rapidly proliferated but also up-regulated markers of activation including CD44, KLRG-1, CD25 and PD1 (Fig 2B). Despite its lag in cell expansion, donor cells responding to the variant virus also up-regulated these activation markers although to a lesser degree in the case of PD-1 and CD25. By day 8 post-infection, cells responding to both viruses had similar levels of CD44 and KLRG-1 while maintaining disparate expression of CD25 and PD-1. Even with evidence of activation, the response to the 35A variant virus resulted in significantly less accumulation of donor CD8+ T cells as compared to infection with the Clone 13 (Fig 2A). While many viral escape mutants are thought to destroy epitope recognition by mutating targeted residues, this data shows that T cells recognize 35A epitope but are sub-optimally activated leading to an inability to maintain the initiated response. The extent of this activation was further demonstrated by *in vitro* peptide stimulation. By day 3 post stimulation, P14 T cells underwent 2–3 divisions to both the GP33 and 35A peptide (Fig 2C). As observed in the *in vivo* model, cells primed with wildtype GP33 peptide expand and rapidly lose CFSE fluorescence while proliferation of variant-primed cells fail to accrue a fully divided population after the initial 2–3 rounds of division. By day 6, there is a dramatic loss in cells responding to the 35A mutant peptide (Fig 2D). Despite this abrogated proliferation, phenotypic analysis shows that 35A variant primed cells up-regulate markers of activation in response to 35A stimulation (Fig 2E). Comparable levels of CD69, CD44, CD25, CD122 and Ki67 are expressed in T cells responding to both GP33 and 35A at 24 hours post stimulation. By day 3, 35A stimulated cells have a slightly less activated phenotype than its wildtype counterpart. By day 6, the disparity between the wild-type response and the variant response is more evident as variant primed cells are unable to sustain their activation response to antigen from day 3 to day 6.

### 35A Stimulation results in reduced viability

**Fig 3:**
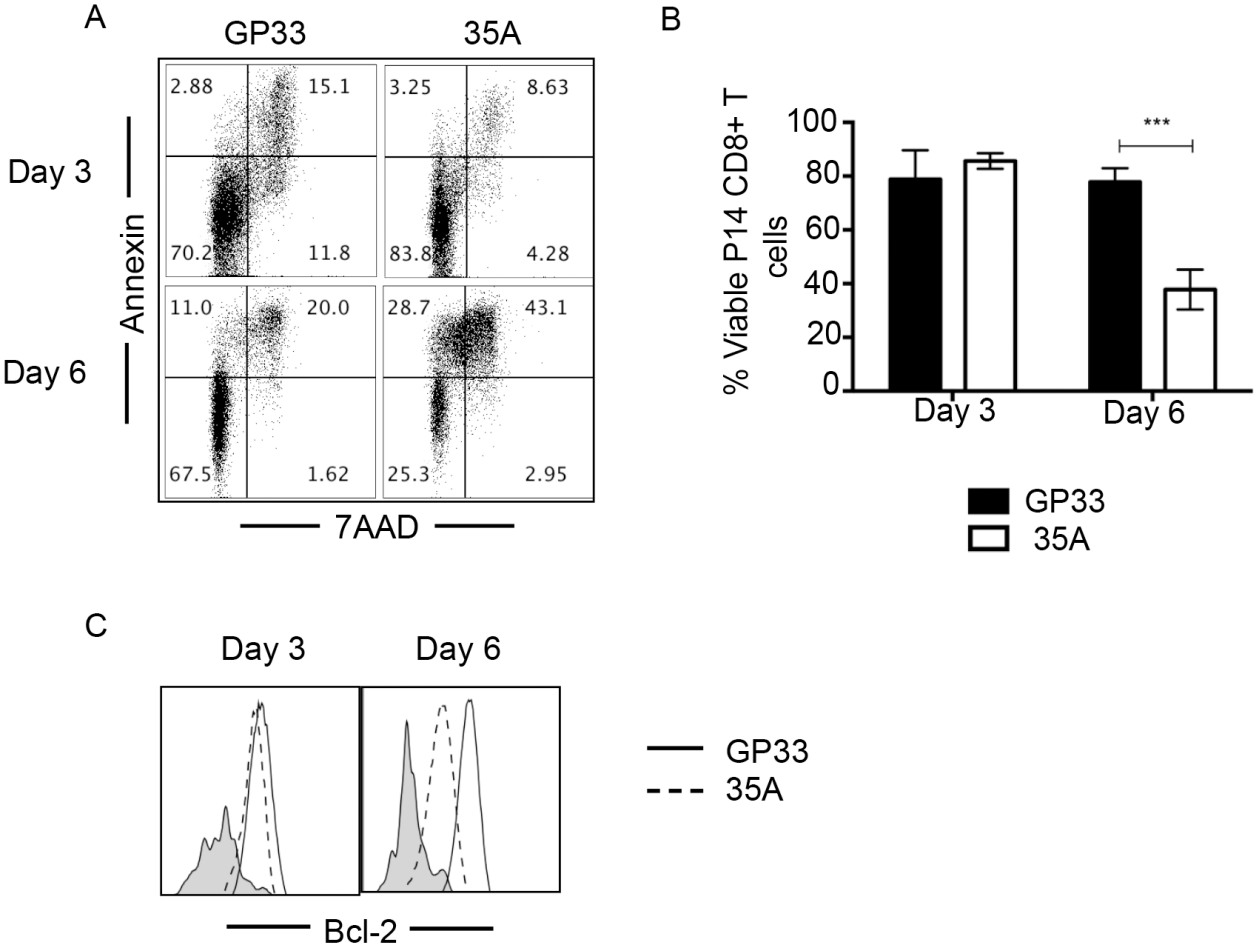
35A Stimulation results in reduced viability in responding cells. P14 CD8+T cells were stimulated with 10μm of GP33 or 35A peptide *in vitro* and evaluated for viability at day 3 and 6 post stimulation by staining with Annexin and 7AAD (A) or using Zombie Yellow cell viability dye (B). Representative flow plot shown in (A). Total of 4 experiments. ***p = 0.002 (Multiple T tests). Error bars indicate ± SEM. Cells were also assayed for the detection of pro-survival Bcl-2 molecule (C) Representative flow plot shown. Total of 2 experiments. Gray shaded histograms refer to unstimulated cells.

To investigate whether the stagnation of cell turnover in response to the 35A epitope was specifically due to death of the responding cells, we assayed for evidence of apoptosis. At day 3, cells stimulated with GP33 and 35A *in vitro* displayed comparable levels of viability as shown by staining with Annexin V and 7AAD (Fig 3A and 3B). However by day 6, less than 40% of the cells responding to the variant were viable (in comparison to approximately 80% viability in response to GP33). Stimulation with 35A is insufficient to maintain pathways that would support viability as we also detected lower levels of the pro survival molecule Bcl-2 at day 3 post 35A stimulation (Fig 3B). This supports the conclusion that suboptimal recognition of the viral variant epitope aborted activation by favoring apoptosis over survival.

### Response to variant characterized by low CD25 expression and deficient IL-2 and IFNγ production

**Fig 4:**
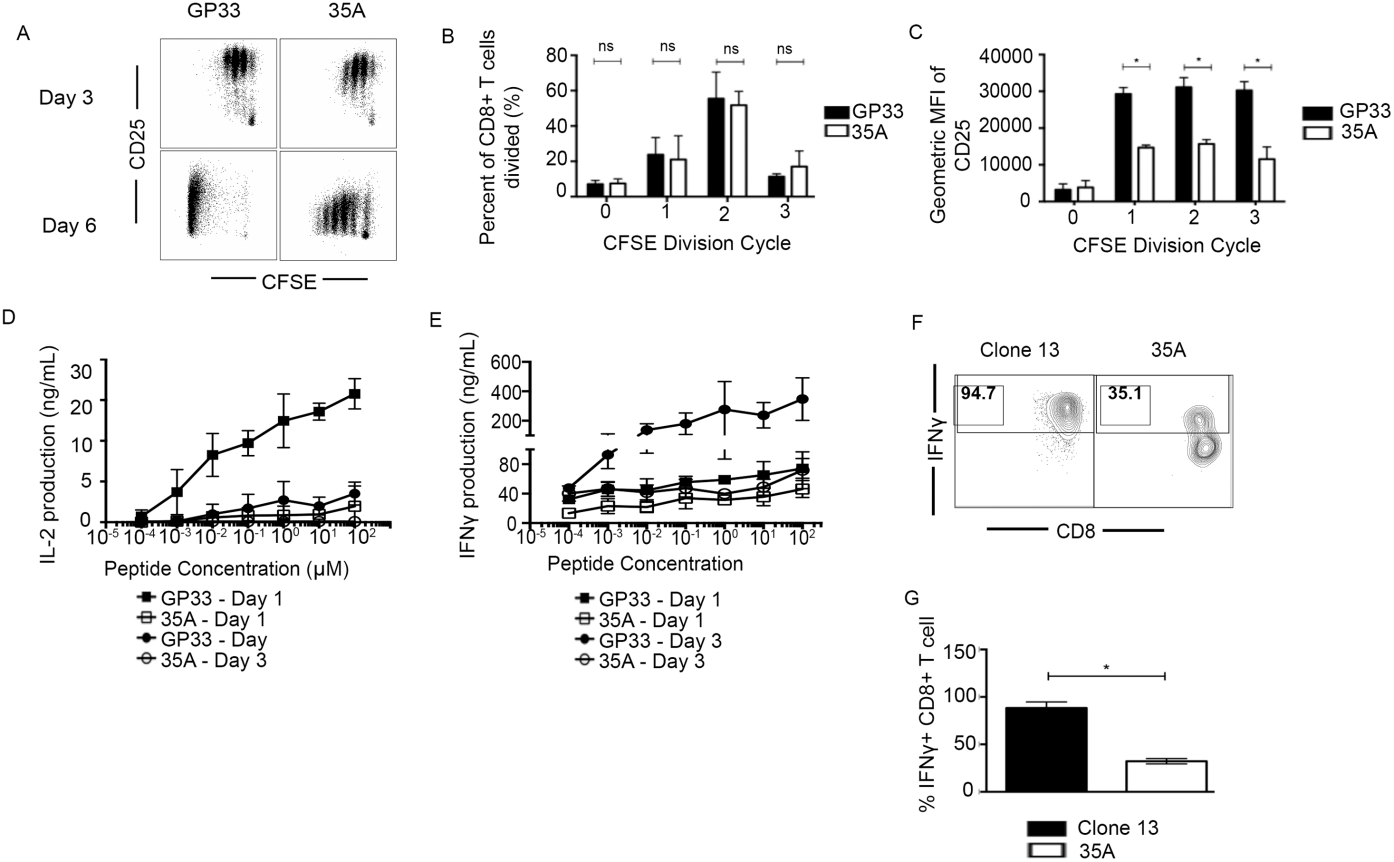
CD25 expression, IL-2, and IFNγ are decreased after 35A stimulation despite equivalent early rounds of division. (A) CD25 expression was assayed on P14 T cells after 3 and 6 days of *in vitro* stimulation with 10μm peptide and correlated with division cycles indicated by CFSE. (B) and (C) Graphical representation of Fig 3A. Average of 2 experiments. *p < 0.05 (Two way ANOVA-Holm Sidak). (D) IL-2 production after 24 and 72 hours of *in vitro* stimulation with peptide measured with ELISA. Average of 3–4 experiments with samples run in duplicate for each time point. (E) IFN**γ** production after 24 and 72 hours of in vitro stimulation with peptide measured via ELISA. Average of 2 experiments with samples run in duplicate for each time point. (F) Representative flow plot of *in vivo* IFN**γ** production at day 4 post infection. (G) Graphical representation of *in vivo* IFN**γ**. Average of 2 experiments with 3 mice pooled for each sample. *p = 0.0153 (Unpaired t test). Error bars indicate ± SEM.

T cells have several requirements for optimal activation, expansion, acquisition of effector function and survival and many are essential in the early stages of T cell activation. In particular, IL-2 is an important cytokine needed for both paracrine and autocrine support of cellular proliferation and survival [28–30]. In addition to decreased survival and proliferation, we noted that cells responding to 35A *in vitro* express lower levels of the high affinity IL-2 receptor, CD25 (Fig 4A). While an equal percentage of wildtype or 35A-stimulated cells had undergone at least 2–3 divisions at day 3, cells responding to 35A in each cycle demonstrated consistently lower CD25 expression in each division cycle (Fig 4B and 4C). Given that CD25 expression is regulated in part by IL-2 levels in an immune microenvironment [31], we next investigated IL-2 production by P14 CD8+ T cells following stimulation with either GP33 or 35A peptide. When we assayed IL-2 production after 24 hours of stimulation, GP33-stimulated effectors produced high levels of IL-2, up to 15ng/mL (Fig 4D). In contrast, we found a severe deficit in the ability of 35A stimulated cells to produce IL-2, as the cytokine was nearly undetectable in the supernatant of cultures stimulated with a range of doses of the variant peptide (Fig 4D). This defect in IL-2 secretion can be seen out to day 3 post peptide stimulation demonstrating that cytokine production is absent and not delayed. We also noted that *in vitro* 35A primed cells were unable to produce the effector cytokine IFNγ at levels comparable to GP33 (Fig 4E), further supporting the idea that mutant epitope recognition initiates an early proliferative program but later results in incomplete acquisition of effector function and phenotype. We also assessed total IFNγ CD8+ T cells directly *ex vivo*. As there were limited numbers of 35A expanded cells to assay cytokine with a complete peptide dose response curve, we chose to stimulate the T cells using phorbal myristate acetate (PMA) and ionomycin. While low cell recovery in 35A infected animals prevented us from adequately assessing the IFNγ response at day 8, we found significantly fewer IFNγ + P14 T cells in 35A infected mice at day 4 post infection (Fig 4F and 4G). This data corroborates our findings in our *in vitro* model that suboptimal recognition of an MHC variant detrimentally impacts the ability of responding T cells to produce cytokine.

### 35A stimulated cells fail to meet metabolic demands for activation and proliferation

**Fig 5:**
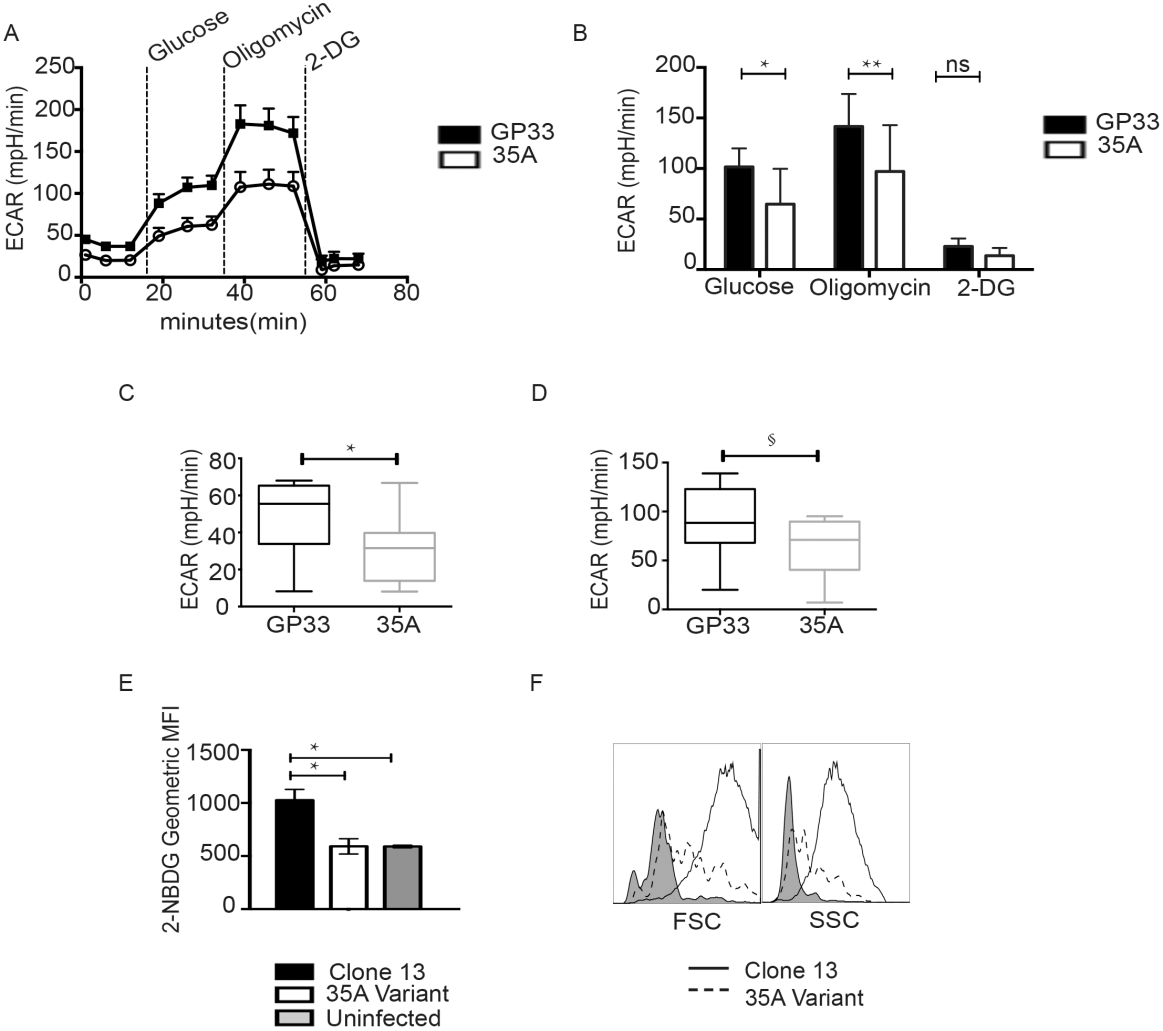
Variant 35A stimulated cells demonstrate a decrease in metabolic fitness. P14 CD8+ T cells were assayed for glycolytic function using the Seahorse extracellular flux assay. (A) Glycolysis stress test assay was performed by sequentially adding the indicated reagents to purified CD8+ cells primed *in vitro* with either GP33 or 35A at day 3. Representative data shown (B) Graphical representation of stress test assay. * p < 0.05, **p < 0.01 (Two way ANOVA-Holm Sidak test). Data shown is the average of 8 experiments with samples run in quadruplicate. Glycolysis (C) and glycolytic capacity (D) were calculated from stress test ECAR values. Glycolysis was calculated by measuring the change in ECAR after addition of glucose and subtracting the basal ECAR levels. Glycolytic capacity is defined as difference between the maximum ECAR achieved after addition of oligomycin and basal ECAR. *p< 0.05, ^§^p = 0.07 (Wilcoxon matched pairs signed rank test) Data shown is the average of 8 experiments with samples run in quadruplicate. (E) Glucose uptake was measured *ex vivo* from infected mice through the detection of fluorescent 2-NBDG by flow cytometry. Two independent experiments with 3 pooled mice in each experiment. Multiple t tests *p < 0.05 (F) *Ex vivo* cells were also analyzed for cell granularity (SSC) and size (FSC) using flow cytometry. Representative data shown. Error bars indicate ± SEM. Gray shaded histograms refer to cells isolated from uninfected control hosts or unstimulated cells.

Lymphocytes require efficient means of generating energy to support the enormous demands of proliferation, effector function and growth [32]. Naïve T cells rely on oxidative phosphorylation to support basal cell functioning. Upon activation however, T cells undergo a glycolytic switch characterized by an increased reliance on glycolysis to generate the ATP and other biosynthetic precursors needed for cellular function and growth [32, 33]. In addition to being important for T cell proliferation and survival, recent work demonstrated this glycolytic switch is critical for the production of IL-2 and IFNγ [34]. As proliferation and cytokine production were compromised in variant stimulated cells, we hypothesized that an altered metabolic state may be a contributing factor. To determine whether cells responding to variant 35A had defects in metabolism we utilized an extracellular flux analyzer to measure glycolysis in cells stimulated with peptide following sequential exposure to glucose, oligomycin and 2-deoxyglucose (2-DG) (Fig 5A and 5B). We consistently found variant primed cells had a lower rate of glycolysis, which was measured by the lactate-induced change in media acidity after the addition of glucose. The addition of the ATP synthase inhibitor, oligomycin, halts oxidative phosphorylation and forces the cell to perform at peak glycolytic capacity which is demonstrated by an increase in the extracellular acidification rate (ECAR) and calculated by subtracting basal ECAR measurements. We observed a significant decrease in glycolysis (Fig 5C) and a decrease in glycolytic capacity (Fig 5D) in CD8+ T cells stimulated with the variant epitope. These parameters of metabolic function are measures of cellular capacity to sustain continued expansion and acquisition of effector function via glycolytic metabolism. As limited T cell numbers prevented us from using the Seahorse based metabolic extracellular assay on *ex vivo* cells, we tested glycolytic function by using the 2-NBDG uptake assay [35]. This assay utilizes a fluorescent glucose analog 2-NBDG which is modified such that it can be taken up by glucose transporters but cannot be fully metabolized. P14 T cells from 35A variant infected mice demonstrated a defect in uptake of 2-NBDG (Fig 5E). In addition, we also observed both decreased size and granularity in 35A-stimulated cells (Fig 5F), which has been shown to correlate with lower rates of glycolysis and reflects a more quiescent metabolic state [36]. Despite the identical 2D affinity and initial TCR engagement, lower pMHC stability affects the ability of variant stimulated cells to undergo glycolysis and thus meet the bioenergetic demands required for sustained proliferation and effector function.

## Discussion

Viral immune evasion from CD8+ T cell responses can hamper effective viral clearance and development of sterilizing immunity. Thus, understanding the mechanisms underlying viral escape can inform the development of effective interventions. The focus of our model is a variant virus containing a single amino acid mutation in the D^b^/GP33 epitope of lymphocytic choriomeningitis virus (LCMV). Infection with the variant 35A virus fails to induce a detectable D^b^/GP33 response and previous studies concluded effector CD8+ T cells were unable to recognize the epitope due its diminished affinity for MHC [16]. However, we demonstrate that P14 CD8+ T cells can initiate several rounds of early division in response to 35A before undergoing a dramatic contraction. In our characterization, we found that variant 35A primed cells exhibited several deficiencies that contributed to the inability of CD8+ T cells to maintain a proliferative response. We observed a severe lack in IL-2 and IFNγ production along with a diminished capacity to undergo glycolytic metabolism. This is an intriguing phenotype as despite having similar affinity for TCR as the wildtype GP33 epitope, T cells have lower and altered IRF4 expression kinetics in response to the variant epitope revealing that 35A is interpreted as a suboptimal TCR stimulus. Our observations define a viral escape method driven by suboptimal triggering of TCR rather complete evasion of the CD8 + T cell response.

Optimal T cell recognition of cognate antigen is essential for achieving full activation of a T cell. Based on current models of T cell activation, peptide:MHC must be of sufficient antigenic quality and require sufficient interaction time to induce downstream signals for initiation of proliferation and cytokine production [3, 37–39]. Though CD8+ T cells need a remarkably short period of engagement with peptide:MHC to distinguish between ligands of similar TCR binding kinetics (< 3 min) and less than 2 hours to initiate a proliferative response [40, 41], T cells require extended time (~40 hours), in conjunction with co-stimulation and cytokine to acquire the properties of an effector cell [42, 43]. For instance, T cell-DC interactions occur in phases that are associated with the acquisition of distinct effector phenotypic qualities [44]. The stability of the peptide:MHC interaction affects the kinetics of the transition through these phases as unstable peptide:MHC complexes prevented long-lasting T cell-DC interactions and optimal T cell responsiveness. Models of T cell activation also rely on TCR affinity as a predictive metric of functional outcome. While TCR affinity for 35A is not different from the native GP33 epitope, 35A has a significantly shorter half-life on H-2D^b^ (Fig 1A). The decreased expression of IRF4 on 35A stimulated cells demonstrates that peptide stability on MHC causes TCR perception of antigen quality to be lower than would be predicted by TCR affinity (Fig 1D–1H). Interestingly, the identical levels of IRF4 at 24 hours in the *in vitro* response suggests 35A is sufficient to initially produce a quality response but the subsequent decrease in IRF4 seen at day 3 indicates the substandard nature of the ligand. Therefore, IRF4 is not simply a direct readout for TCR affinity for antigen, but it can also readout overall quality and strength of the TCR interaction with pMHC ligand, which in this case is greatly affected by the shorter half-life of 35A. While both wildtype GP33 and variant 35A can initiate 2–3 early rounds of preprogrammed cell division by day 3 post stimulation (Fig 2C), the proliferation of 35A-primed cells rapidly diminishes thereafter resulting in less cell accumulation at later time points (Fig 2A and 2D), day 8 *in vivo*, day 6 *in vitro* respectively. Our previous characterization of the intracellular signaling response to the variant epitope demonstrated deficient downstream intracellular signaling as evidenced by low pErk, low Egr1 expression and sustained SHP-1 activity which blocks further positive signaling downstream of the TCR [17]. Indeed, recent work has shown that T cells need as little as 3 minutes to interpret differences between closely related peptides and initiate an appropriate response based on signaling cascades engaged [45]. Therefore while some downstream propagation of TCR signaling occurs, variant peptide stimulation provides insufficient signal to support continued clonal expansion.

While IL-2 has been found to be expendable for the earliest rounds of division, maintenance of the proliferative response is IL-2 dependent [30]. We observed a severe deficit in the ability of 35A primed T cells to produce IL-2 (Fig 4D), as well as decreased expression of the high affinity IL-2Rα chain CD25 on cells responding to the variant (Fig 4A). In addition, the CD25 expression inversely correlated with number of division cycles (*in vitro*), resulting in fewer variant primed cells entering later division cycles (Fig 4C). Our data shows that while 35A recognition meets the minimum threshold of activation to engage this “auto-proliferative” response, the response lacks sufficient signals to maintain an activated phenotype. Diminished production of IFNγ both in vitro (Fig 4E) and *in vivo* (Fig 4F and 4G) further highlights that variant cells are unable to completely acquire effector function.

The consequence of this low quality TCR stimulation by 35A is a significant increase in apoptosis, as evidenced by co-staining with Annexin V and 7AAD and diminished expression of the anti apoptotic protein Bcl-2 in 35A stimulated cells (Fig 3). Low Bcl-2 levels are indicative of skewing towards apoptotic signaling as T cells balance both Bcl-2 mediated pro-survival and Bax/Bim mediated pro-apoptotic death signals [46, 47]. Several studies have highlighted the ability of T cells to mount a response to low affinity antigens with the outcome being a contracted or restricted response mediated by decreased division cycles of responding cells and/or increased apoptosis [48–50]. However, in those studies, the variant had mutations at TCR contact residues that directly modulated TCR affinity. Here, we demonstrate that an epitope with reduced affinity for MHC rather than TCR can also negatively modulate the T cell response by inducing division and subsequent death of dividing cells. The study of MHC variants is particularly impactful as this type of mutational escape has the potential to effect a wider breadth of CTL responses, both on an individual and population level, than if the mutation affected a single TCR clone as APLs do. Additionally, it has been observed that the majority of escape mutations identified in HIV infections affect binding to HLA [51, 52]. Thus our model is particularly relevant for studying differences in how disruption of MHC binding to viral antigens differ from that of TCR binding to MHC:peptide and how that may affect development of therapeutic and prophylactic interventions.

Recent work has highlighted the importance of cellular metabolism in modulating T cell fate decisions [32, 33, 36, 53]. The transition from a naïve to an activated CD8+ T cell is associated with metabolic reprogramming to a glycolytic phenotype. Additionally, several studies have connected the ability of T cells to produce cytokines with its mode of generating metabolic fuel [34, 54]. Although switching from mainly oxidative phosphorylation to aerobic glycolysis is important for sustaining proliferation and survival, it is most critically required for cytokine production. Since we observed a deficit in both IL-2 and IFNγ production in our 35A stimulated cells, we investigated whether metabolic differences could further explain the defective CD8+ T cell response to variant 35A at later time points. Our results indicated that variant-primed cells were lacking in their ability to undergo glycolysis and also were inefficient in glucose uptake (Fig 5). This may be due to less glucose receptor expression and/or inability to efficiently metabolize glucose into the lactate end product. As aerobic glycolysis is important for generation of ATP as well as secondary byproducts used for synthesis of lipids, nucleotides and other proteins, the various metabolic defects in variant primed cells provide an explanation for why they fail to thrive later in the response. It should also be noted that IRF4 expression has been found to regulate transcription of enzymes involved in metabolic function like glucose transport receptors and HIF-1, accordingly [26]. Our observation showed IRF4 correlated with both peptide dose and peptide potency, with the variant response found to have the lowest IRF4 expression (Fig 1). Based on these observations, it is plausible that lower IRF4 expression in variant primed cells may contribute to the inability of the cells to efficiently produce the glycolysis-derived energy needed to fuel proliferation and survival.

Targeting the metabolic state of immune cells for therapeutic purposes has shown great promise in models of viral infection, cancer, and autoimmunity [55, 56] Our results found that T cells responding to the variant 35A have decreased glycolysis and glycolytic capacity as compared to the response to the wildtype epitope. We might then hypothesize that therapy to enhance glycolysis may allow for increased survival of T cells confronted with MHC variants. It should be noted that other studies have found that blocking glycolysis with 2-deoxyglucose (2-DG) augments the formation of memory CD8+ T cell responses while enhancing glycolysis led to an increase of short term CD8+ effectors during primary response [36]. It has also been shown that glycolytic flux is essential for the acquisition of IFNγ producing abilities, particularly by memory CD8+ T cells undergoing secondary re-challenge [34, 57]. Thus, therapies to modulate T cell metabolic programs require careful consideration of the target cell population.

In conclusion, this study demonstrates that immunological ignorance is not simply a passive form of viral evasion, but rather an active process by which viral variants disrupt optimal T cell recognition leading to the deletion of activated effectors through modulation of critical pathways of T cell survival and activation. Recovery of the response to viral variant may require a multi-targeted approach involving both increasing cellular perception of signal strength and modulation of the bioenergetic program.

## Materials and Methods

### Mice

P14 CD45.1 or P14 Thy 1.1 TCR transgenic mice [58] were housed and bred in the Emory University Department of Animal Resources facility and used in accordance with the Institutional Animal Care and Use Committee—approved protocols. C57BL6 mice used as hosts for adoptive transfers were purchased from National Cancer Institute (NCI) or The Jackson Laboratory. Mice were used between 8–15 weeks of age.

#### Ethics Statement

This study was carried out in strict accordance with the recommendations in the Guide for the Care and Use of Laboratory Animals of the National Institutes of Health. The protocol was approved by the Emory University Institutional Animal Care and Use Committee (IACUC) Approval number: DAR-2002722-051317GN. All intravenous injections and euthanasia techniques (CO_2_ exposure) were done according to the approved IACUC protocols and all efforts were made to minimize undue suffering. Mice were monitored daily for signs of adverse health effects (reduced grooming, excessive weight loss, lethargy)

### Peptides

Peptides were synthesized at Emory University on a Peptide Technologies Incorporated Prelude synthesizer. The GP33-41 sequence (KAVYNFATM) and the 35A variant sequence (KAAYNFATM) were used at a concentration of 10μm unless otherwise indicated.

### Virus

Lymphocytic choriomeningitis virus (LCMV) Clone 13 and variant 35A were kind gifts from Dr. Rafi Ahmed (Emory University) and were made as described in [59, 60]

### Adoptive Transfers

CD8+ T cells were purified from P14 TCR transgenic animals using the EasySep CD8+ T cell Enrichment Kit (StemCell) and transferred intravenously to congenic C57Bl/6 hosts. Mice were intravenously infected with 2x10^3^ PFU of either virus 24 hours after adoptive transfer. Spleens were harvested at indicated time points for analysis of donor cell expansion and phenotype.

### Cell Culture

P14 splenocytes were stimulated for one hour with peptide and then washed and re-plated in 96 well plates. For analysis of CFSE division, cells were labeled with 2.5mM CFSE for 3 minutes prior to peptide stimulation. Cells were subsequently incubated with fetal bovine serum (FBS) then washed with media. Cells were then cultured in 37°C incubator for indicated time periods after which cells were harvested for phenotypic analysis.

### Enzyme-linked immunosorbent assay (ELISA)

To measure IL-2 and IFNγ, supernatants from cell cultures were added to plates previously coated with purified anti-IL 2 or anti- IFNγ antibody. After overnight incubation, biotinylated anti-IL2 or anti- IFNγ antibody (Ebioscience) were added followed by alkaline phosphatase-conjugated avidin and p-nitrophenylphosphate substrate (Sigma). Cytokine concentrations were determined based on standard curve derived from recombinant stocks of IL-2 and IFNγ.

### Flow cytometry

Cells were stained on ice with antibodies to surface markers (CD25 (PC61), CD44 (IM7), KLRG1 (2F1), PD1(29F.1A12), CD69 (H1.2F3), CD122 (TM-b1), H-2D^b^ (28-14-8) THY1.1 (HIS51) for 30 minutes on ice. For detection of intracellular markers (Bcl-2 (3F11), IRF4 (3E4), Ki67 (B56), IFNγ (XMG1.2)), cells were fixed and permeabilized using the Transcription Factor Staining Set (Ebioscience). Annexin V, 7AAD and Zombie Yellow Viability Kit (Biolegend) were utilized according to manufacturers instructions. All antibodies were purchased from BD or Ebioscience.

### Metabolic Assays

For the 2-NBDG uptake assay, cells were re-suspended in glucose free media and allowed to incubate with 10μm of 2-NBDG for 20 minutes. Cells were washed with glucose free media and analyzed by flow cytometry. The glycolysis stress test was performed by purifying CD8+ T cells using the EasySep CD8+ T cell Enrichment (StemCell) and adhering them to a 96 well plate with CellTak tissue adhesive (Corning). Cells were incubated in a CO_2_ free incubator for a minimum of 30 minutes. Samples were then run on the Seahorse XF extracellular analyzer 96^e^ where glucose, oligomycin and 2-deoxyglucose (2-DG) were sequentially added to cells. Changes in glycolytic activity were measured by changes in extracellular acidification rate (ECAR). Glycolysis was calculated by measuring the change in ECAR after addition of glucose and subtracting the basal ECAR levels. Glycolytic capacity is defined as the difference between maximum ECAR achieved after addition of oligomycin and basal ECAR levels.

### Two-dimensional (2D) micropipette adhesion frequency assay

Human RBCs (obtained from healthy volunteers) were biotinylated using Biotin-X-NHS (EMD4 Biosciences) and subsequently labeled with streptavidin (Thermo Scientific) and biotinylated peptide:MHC-I monomers. P14 T cells were purified from spleens using the EasySep CD8+ T cell Enrichment Kit (Stemcell). Single cells were aspirated onto opposing glass micropipettes in a cell chamber mounted on the stage of an inverted light microscope. An electronically controlled piezoelectric actuator controlled repeated T cell contact with a stationary pMHC-coated RBC for two seconds. Binding events were visualized as an elongation of the RBC membrane upon retraction of the T cell. Cells were brought into contact 50 times in order to calculate an adhesion frequency (P_a_). Quantification of pMHC ligand (m_l_) and TCR-α receptor (m_r_) densities were determined by flow cytometry using BD QuantiBRITE PE Beads for standardization (BD Biosciences). Surface densities as well as adhesion frequency (Pa) were then used to calculate two-dimensional affinity using the following equation: A_c_K_a_ = -ln[1-P_a_(∞)]/m_r_m_l_. Geometric mean affinities are reported ± SEM. This assay is also described in detail in [18] and [61]

### RMA/S MHC Class I Stability Assay

RMA/S cells were incubated at room temperature overnight to allow for surface expression of empty H-2D^b^. Cells were plated at 2x10^5^ cells per well in a flat bottom 96 well plate and loaded with peptides of interest. Cells were placed at 37°C to induce downregulation of unoccupied MHC molecules and harvested at indicated time points to assay for expression of H-2D^b^ by flow cytometry. Half life was calculated by using data from Fig 1C and is expressed in the following equation: t_1/2_ = (t*ln(2))/ln(N_0_/N_f_,) where t_1/2_ = half life, t = time elapsed, N_0_ = initial H-2D^b^ expression after loading, N_f_ = final H-2D^b^ expression at final time point assayed.

### Statistical Analysis

Statistical analyses were performed using GraphPad Prism 6 software (Software for Science). Significance was calculated using two-way ANOVA, unpaired t test, and Wilcoxon matched pairs signed rank tests.
